# Structure and Correlates of Cognitive Aging in a Narrow Age Cohort

**DOI:** 10.1037/a0036187

**Published:** 2014-06

**Authors:** Elliot M. Tucker-Drob, Daniel A. Briley, John M. Starr, Ian J. Deary

**Affiliations:** 1 Department of Psychology and Population Research Center, University of Texas at Austin; 2 Centre for Cognitive Ageing and Cognitive Epidemiology, Department of Psychology, University of Edinburgh, Edinburgh, Scotland; Alzheimer Scotland Dementia Research Centre, Department of Psychology, University of Edinburgh; 3 Centre for Cognitive Ageing and Cognitive Epidemiology, Department of Psychology, University of Edinburgh

**Keywords:** cognitive aging, common cause hypothesis, latent difference score model, longitudinal change

## Abstract

Aging-related changes occur for multiple domains of cognitive functioning. An accumulating body of research indicates that, rather than representing statistically independent phenomena, aging-related cognitive changes are moderately to strongly correlated across domains. However, previous studies have typically been conducted in age-heterogeneous samples over longitudinal time lags of 6 or more years, and have failed to consider whether results are robust to a comprehensive set of controls. Capitalizing on 3-year longitudinal data from the Lothian Birth Cohort of 1936, we took a longitudinal narrow age cohort approach to examine cross-domain cognitive change interrelations from ages 70 to 73 years. We fit multivariate latent difference score models to factors representing visuospatial ability, processing speed, memory, and crystallized ability. Changes were moderately interrelated, with a general factor of change accounting for 47% of the variance in changes across domains. Change interrelations persisted at close to full strength after controlling for a comprehensive set of demographic, physical, and medical factors including educational attainment, childhood intelligence, physical function, *APOE* genotype, smoking status, diagnosis of hypertension, diagnosis of cardiovascular disease, and diagnosis of diabetes. Thus, the positive manifold of aging-related cognitive changes is highly robust in that it can be detected in a narrow age cohort followed over a relatively brief longitudinal period, and persists even after controlling for many potential confounders.

In the general population of older adults, aging-related declines are well documented for a variety of domains of cognitive function (Harris & Deary, 2011). Although such declines are normative, there is nevertheless notable between-persons heterogeneity in rates of decline, with some individuals evincing comparatively little change and others evincing dramatic change over time (Tucker-Drob & Salthouse, 2011). An important question, then, has been how these individual differences interrelate across cognitive domains (Tucker-Drob, 2011a, 2011b): Do individual differences in cognitive decline reflect synchronous within-person changes across a broad range of cognitive domains (potentially structured along a single common dimension), or are individual differences in change independent across domains, with some individuals declining more dramatically in some cognitive domains and others declining more dramatically in others? Rabbitt (1993) phrased this research question succinctly when he asked, “Does it all go together when it goes?” (p. 385). Furthermore, to the extent that synchronous within-person changes across different domains occur, can these interrelations be attributed to “third variable effects” of demographic characteristics, physical constitution, or medical or other risk factors?

Two major methodological impediments have historically made addressing these basic questions difficult. First, because single-occasion data can be collected relatively easily from large age-heterogeneous samples during a short period of time, the overwhelming majority of research within the field of cognitive aging has typically employed cross-sectional approaches. These approaches are well suited for investigating patterns of mean age-related differences within a multivariate system of cognitive variables (see, e.g., Salthouse, 2004). However, cross-sectional data are not directly informative about the dimensionality of longitudinal changes. For instance, using formal mathematical proof, Lindenberger, von Oertzen, Ghisletta, and Hertzog (2011; also see Hofer, Flaherty, & Hoffman, 2006) concluded that

Given its brittle and volatile link to correlated change, [the cross-sectional mediation approach] is more of a hindrance than a help in the quest to delineate the temporal ordering and causal structure of behavioral change . . . . It is generally not known whether multivariate structures based on between-person differences are valid approximations to the structure of change within a given individual. (p. 40)

Second, even when longitudinal data are available, conventional approaches to estimating or factor analyzing correlations among individual differences in change have methodological problems that severely bias results. As Cronbach and Furby (1970) warned over 40 years ago, “‘Raw change’ or ‘raw gain’ scores formed by subtracting pretest scores from posttest scores lead to fallacious conclusions, primarily because such scores are systematically related to any random error of measurement” (p. 68).

Only recently have researchers begun to examine the interrelations among, and structure of, aging-related longitudinal cognitive changes by applying statistical approaches that are capable of eliminating bias by separating systematic changes from unsystematic sources of error (McArdle & Nesselroade, 2003). Such statistical approaches can generally be classified as growth curve models (which can be specified using structural equation modeling, multilevel modeling, random effects modeling, and hierarchical linear modeling frameworks) and latent difference score models (which are typically specified using a structural equation modeling framework, as was the case for the current project). Results from all such major studies that we have been able to identify are summarized in Table 1. It can be seen that individual differences in longitudinal cognitive changes have consistently been found to be moderately interrelated, with a common factor accounting for between approximately 30% and 70% of the variance. Samples have tended to be composed of middle-aged and older adults, with only a few studies including younger adults, and all samples included a fairly broad range of ages rather than a single narrow age cohort, with maximum longitudinal time lags ranging between approximately 4 and 20 years. Finally, it is of note that the studies listed have examined control variables to a very limited extent, generally limiting themselves to age, retest interval, and dementia status.[Table-anchor tbl1]

Here, we build on previous research in a number of important respects. First, we analyzed data from a single narrow age cohort of 70-year-old individuals (ages 67.7–71.4 years at baseline, *SD* = 0.83). By minimizing age heterogeneity in our sample, we ensured that nearly all changes observed reflect individual differences in change over time rather than age-related differences in levels of performance or the magnitude of longitudinal change (Hofer & Sliwinski, 2001). This is of particular note because many previous studies have employed age-based growth modeling of longitudinal data, which blends longitudinal information regarding within-person changes with cross-sectional information regarding between-persons differences. Second, we examined changes over a relatively short duration: approximately 3 years of aging (range = 1.8–4.8 years, *SD* = 0.28). Detecting interrelations among variables over this relatively short period of time would speak to the robustness of the phenomenon of correlated change. Third, we examined whether interrelated changes persist after controlling for a number of key variables previously implicated in differences in age-related cognitive decline, including age, time interval, early life intelligence, educational attainment, and a number of key indices of physical and mental health (for a systematic review of evidence for putative risk and protective factors for cognitive decline in older adults, see Plassman, Williams, Burke, Holsinger, & Benjamin, 2010). If the correlations among longitudinal changes are robust to these controls, this would indicate that they are not artifacts or epiphenomena of different abilities being predicted by the same set of risk and protective factors (Baltes, Nesselroade, & Cornelius, 1978).

## Method

### Participants

Data were derived from the Lothian Birth Cohort of 1936 (LBC1936) study, which began tracking 1,091 independently living Scottish adults between 2004 and 2008 (i.e., at approximately age 70 years), with the intent to follow them longitudinally (Deary et al., 2007). All were born in 1936. The LBC1936 study was designed to take advantage of the fact that on June 4, 1947, nearly all children born in 1936 and attending school in Scotland sat for a group-administered cognitive ability test (Deary, Whalley, & Starr, 2009). For practical reasons, participants were required to be living in the Edinburgh area, where the LBC1936 study took place. Further details about the tracing, recruitment, testing of, and publications from the LBC1936 study can be found in previous publications (Deary, Gow, Pattie, & Starr, 2012; Deary et al., 2007). Mean age at baseline was 69.58 years (*SD* = 0.83, *n* = 1,091) and mean age at follow-up was 72.54 years (*SD* = 0.71, *n* = 866). Mean longitudinal time lag was 2.98 years (*SD* = 0.28, *n* = 866). Men composed 50.2% of the sample at baseline and 51.7% at follow-up.

### Longitudinal Cognitive Ability Measures

We constructed latent variables based on three to four indicators of *visuospatial ability*, *processing speed*, *memory*, and *crystallized ability*, which were taken at baseline (age 70) and 3-year follow-up (age 73).

Visuospatial ability was measured with Matrix Reasoning and Block Design from the Wechsler Adult Intelligence Scale—Third Edition (WAIS–III; Wechsler, 1998a) and Spatial Span Forward and Spatial Span Backward from the Wechsler Memory Scale—Third Edition (WMS–III; Wechsler, 1998b). Previous studies (e.g., Johnson & Deary, 2011; Salthouse, Pink, & Tucker-Drob, 2008) have indicated that measures of spatial memory (e.g., Spatial Span) and fluid reasoning (e.g., Matrix Reasoning and Block Design) load strongly on a common underlying dimension of individual differences.

Processing speed was measured with Symbol Search and Digit Symbol from the WAIS–III, and Inspection Time, and Choice Reaction Time. Inspection Time is described in detail in Deary, Simonotto et al. (2004), and Choice Reaction Time is described in detail in Deary, Der, and Ford (2001). In brief, inspection time contains items requiring participants to indicate by unspeeded button press which of two vertical lines is longer, with the stimuli being presented at a number of different durations. Choice Reaction Time requires participants to press labeled buttons that correspond to digits presented (1 to 4).

Memory was measured with Logical Memory, Verbal Paired Associates, and Digit Span Backwards, all from the WMS–III.

Crystallized ability was measured with National Adult Reading Test (NART; Nelson & Willison, 1991), Wechsler Test of Adult Reading (WTAR; Wechsler, 2001), and Verbal Fluency (Lezak, 2004). Both the NART and WTAR involve participants reading aloud lists of words and are scored based on correct pronunciation. Verbal Fluency contains three trials in which participants are asked to name as many words as possible beginning with the letters C, F, and L, respectively, in 1 min per letter.

### Covariates

We selected a number of covariates that have previously been implicated in individual differences in cognitive decline (Plassman et al., 2010) and could therefore potentially account for correlated longitudinal changes across cognitive domains. Broadly, these covariates related to demographics, physical health, and medical risk factors.

Demographic characteristics included age at baseline, longitudinal time lag, childhood intelligence, educational attainment, and sex. Age at baseline and longitudinal time lag were measured as days from birth and days from the initial assessment (and subsequently divided by 365 such that they were scaled in years) and are described above in the Participants section. Childhood intelligence was measured as part of the Scottish Mental Survey of 1947, when participants were age 11 years. This was measured using a group test called the Moray House Test No. 12, which has a preponderance of verbal reasoning items and had a correlation of about .8 with the Stanford–Binet Test (Deary et al., 2009). Educational attainment was self-reported at baseline in terms of years of completed full-time education. Sex was coded as 0 = female and 1 = male.

Physical health included aspects of general health not specifically associated with medical problems. Forced expiratory volume in 1 s was measured with a microspirometer at baseline. Six-meter walk time was measured at baseline as the amount of time taken to walk 6 m at a normal pace, was log transformed to better approximate a normal distribution, and then reflected (by multiplying by −1) such that high scores would indicate faster walking. Grip strength was assessed with a Jamar hydraulic hand dynamometer for the right and left hands three times each. The best strength for each hand were then averaged together.

Medical risk factors known to be associated with cognitive aging were included. *Apolipoprotein E* (*APOE*) genotype was determined based on DNA extracted from whole blood samples collected at baseline. The ε4 allele is implicated as a risk variant for cognitive aging (Harris & Deary, 2011). Individuals with at least one copy of the ε4 allele were coded as 1 (*n* = 306), and individuals without any copies of the ε4 allele were coded as 0 (*n* = 722). Smoking status at baseline was coded as 0 for nonsmokers or former smokers (*n* = 966) and as 1 for current smokers (*n* = 125) based on self-report. Cardiovascular disease status at baseline was coded as 0 for no diagnosis of cardiovascular disease (*n* = 823) and as 1 for an affirmative diagnosis of cardiovascular disease (*n* = 268) based on interview. Hypertension diagnosis was coded as 0 for no diagnosis (*n* = 658) and as 1 for an affirmative diagnosis (*n* = 433) based on interview. Diabetes diagnosis was made based on a combination of self-report of diagnosis and glycated hemoglobin (HbA1c) levels determined from blood samples taken at baseline (World Health Organization, 2011). The variable was coded dichotomously to reflect HbA1c levels less than 6.5% and no self-report of diabetes diagnosis (no diagnosis coded 0, *n* = 922) compared with HbA1c levels greater than or equal to 6.5% or affirmative self-report of diabetes diagnosis (diagnosis coded 1, *n* = 139).

### Analytic Approach

**Figure 1 fig1:**
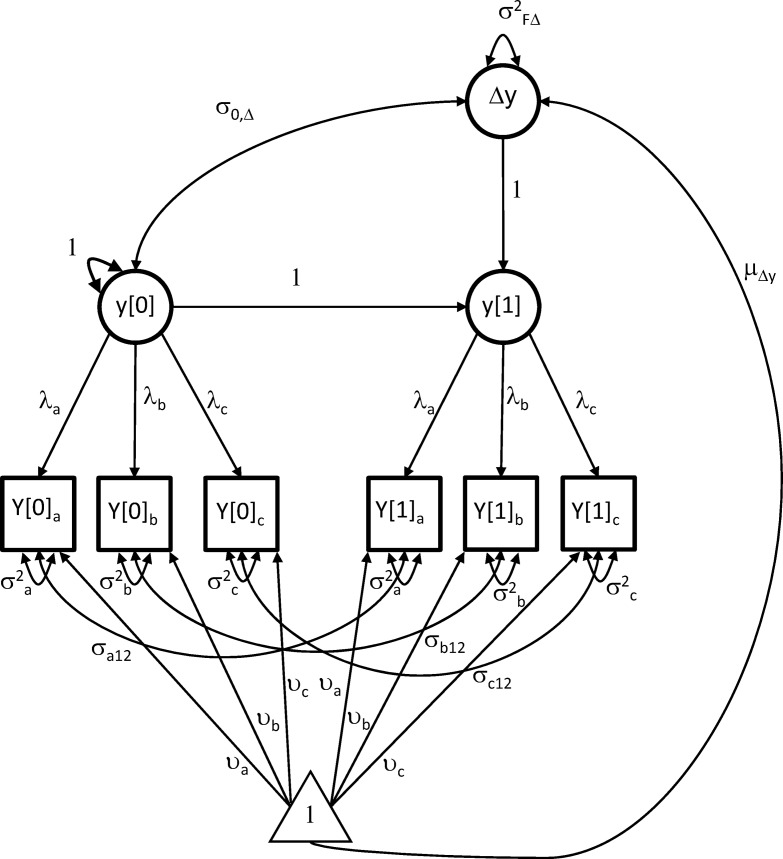
Path diagram for a single factor (*y*) measured by three indicators (*Y*_*a*_, *Y*_*b*_, and *Y*_*c*_) at baseline [0] and follow-up [1] occasions.

We made use of a latent difference score modeling approach (McArdle, 2009), a univariate version of which is represented as a path diagram in Figure 1. The measurement portion of this approach specifies a latent factor, *y*, measured by multiple tests (e.g., *Y*_*a*_, *Y*_*b*_, and *Y*_*c*_) on two occasions separated in time. The brackets [0] and [1] denote baseline and follow-up occasions, respectively. Each test is specified to load on the occasion-specific latent variable with a loading (λ), and each test is allowed to have an intercept (υ) and a residual variance (σ^2^). Cross-time residual autocorrelations (σ_12_) are allowed for each test. The baseline factor is set to the *z*-metric (*M* = 0, *SD* = 1), and the mean and the variance of the difference score can therefore be interpreted relative to this metric.[Fig-anchor fig1]

In the higher order difference score portion of the model, the latent factor at follow-up (*y*[1]) is regressed onto the latent factor at baseline (*y*[0]) at a fixed value of 1, and allowed to have a residual (Δ*y*). This portion of the model is a simple linear regression, written as

y[1]=1⁢×y[0]+Δy.

Rearranging the regression equation demonstrates that the residual (Δ*y*) represents a difference score between the latent factor scores at baseline and follow-up:

Δy=y[1]−y[0].

Note that because the difference score occurs between two latent factors, each of which has been purged of measurement error, it is itself measurement error free. This latent difference score is allowed to have a mean, a variance, and a covariance with the baseline factor score. Covariates can be added to the model, onto which the latent difference score can be regressed.

**Figure 2 fig2:**
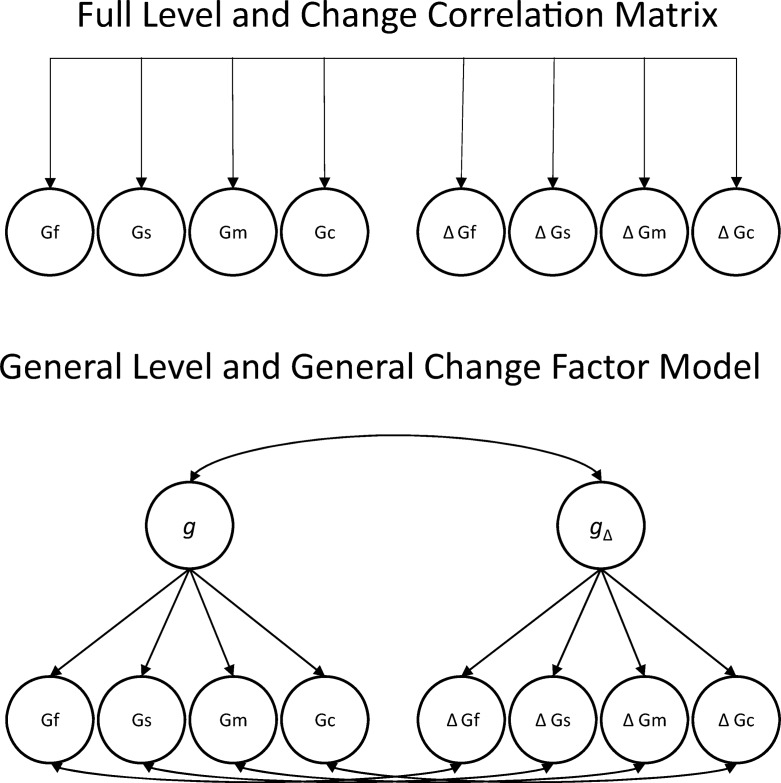
Representation of two complementary approaches to modeling baseline level and change correlations in a multivariate latent difference score model. The top panel represents a model in which all correlations between and among levels and changes are correlated. The bottom panel represents a model in which a common general intelligence (*g*) factor is fit to the baseline levels and a separate common general change (*g*_Δ_) factor is fit to the rates of change. Gv = visuospatial ability; Gs = processing speed; Gm = memory; Gc = crystallized ability; Δ = change. Measurement portions of the models and details (i.e., variances and residual variances, mean structure, and parameter labels) have been omitted for ease of presentation.

In the multivariate version of the latent difference score model depicted in the top panel of Figure 2, relations among baseline factors, among difference scores, and between baseline factors and difference scores for factors representing different cognitive abilities and changes therein are estimated. In the model depicted in the bottom panel of Figure 2, higher order common factors are specified for the baseline factors and the changes. We report results from both of these multivariate approaches in the current article. We also report results for models that do and do not include controls for a host of covariates.[Fig-anchor fig2]

## Results

Structural equation modeling was performed using Mplus Version 7.1 (Muthén & Muthén, 1998–2012). Descriptive statistics for the cognitive variables are presented in Table 2. Measurement invariance of the latent visuospatial, processing speed, memory, and crystallized ability factors was tested using the general procedure described by Widaman, Ferrer, and Conger (2010). We began with a baseline model (configural invariance) in which the same factor loading pattern was specified at baseline and follow-up occasions, and progressively imposed cross-time invariance of factor loadings (weak factorial invariance), intercepts (strong factorial invariance), and residual variances (strict factorial invariance) in successive models. The metric of the common factors at baseline was set to a *z*-scale (*M* = 0, *SD* = 1). To identify the metrics of the common factors at the follow-up occasion, we constrained the loading and intercept of the first indicator of each factor to be equal across time. Fit statistics for the four models used to examine measurement invariance are presented in Table 3. Nested χ^2^ comparisons indicated a significant increase for each model relative to the preceding model. However, the fit of each model was excellent as assessed by root mean square error of approximation (RMSEA), and the model constraints did not worsen RMSEA appreciably. Bayesian information criterion comparisons indicated that strict invariance (equal loadings, intercepts, and residual variances) was preferred. Akaike information criterion comparisons favored configural invariance, but these values were fairly similar across the models. To evaluate potential sources of misfit, we examined the loadings, intercepts, and residual variances of the indicators in the baseline (configural invariance) and final (strict invariance) models. These results are presented in Table 4. As can be seen, there were only very small differences between the loading, intercepts, and residual variances across the two time points in the baseline (configural invariance) model, and the parameters in the final (strict invariance) model that imposed equivalence were very similar to the estimates from the baseline model at both time points. Based on these observations, we chose to adopt strict factorial invariance for all further analyses. [Table-anchor tbl2][Table-anchor tbl3][Table-anchor tbl4]

Focusing on the parameter estimates from the measurement portion of the multivariable latent difference score model, which imposed strict invariance (see the right portion of Table 4), it can be seen in Table 4 that all standardized loadings were moderate to large in magnitude, ranging in absolute magnitude from .45 to .95. The mean and variance of each of the four latent difference scores are presented in Table 5. It can be seen that there was statistically significant variance in each of the latent difference scores, except for visuospatial ability change, for which there was marginally significant variance. It can also be seen that there was significantly negative mean change for all latent difference scores except for memory change, for which the mean change was positive. The positive change in memory is likely attributable to a practice effect driven by participants having some memory or familiarity with the memory stimuli, superimposed over an effect of aging-related memory decline. Importantly, there was substantial variability in the amount and direction of change in memory as indicated by a slope standard deviation nearly 7 times as large as the slope mean. In addition, previous studies have indicated that there are little if any individual differences in the impact of practice effects on test scores (Salthouse & Tucker-Drob, 2008; Tucker-Drob, 2011a). Therefore, we later interpret correlates of individual differences in memory change in terms of a correlate being associated with more or less decline (as opposed to less or more gain).[Table-anchor tbl5]

Correlations among level factors, among latent difference scores, and between levels and latent difference scores are presented in Table 6. Also provided are factor loadings from an alternative model in which, rather than allowing for factor intercorrelations, a higher order general factor of levels and a separate higher order general factor of changes are fit (as in the bottom panel of Figure 2). This model fit well (RMSEA = .044, comparative fit index = .958, Tucker–Lewis index = .956). Consistent with Spearman’s (1904) positive manifold, it can be seen that levels of performance in each of the four abilities were correlated at moderate to large magnitudes, such that when a *g* factor of levels was fit, factor loadings ranged from approximately .70 to nearly .87. An average of 64% of the variance in the levels was accounted for by the *g* factor. However, the *g* factor accounted for a relatively larger amount of variance in visuospatial ability, processing speed, and memory (69% on average) than it did in crystallized ability (50%) by the general factor.[Table-anchor tbl6]

Of particular note is that a similar positive manifold of moderate in magnitude change interrelations was evident. All change intercorrelations were statistically significant, with the exception of the processing speed change–visuospatial ability change correlation, which was marginally significant. When the latent difference scores were specified to load on a common factor of changes (as in the bottom panel of Figure 2), factor loadings ranged from approximately .55 to .83 and all were statistically significant. An average of 48% of the variance in the changes was accounted for by this general change factor. As was the case for the *g* factor of levels, a relatively larger amount of variance was accounted for by the general factor of changes for visuospatial ability, processing speed, and memory (54%) compared with crystallized ability (30%). Furthermore, the average proportion of variance (48%) accounted for by the common change factor was noticeably smaller than the proportion of variance in levels accounted for by the *g* factor (64%), but it was sizable nevertheless, accounting for nearly half of the total variance in change.

Next, we examined whether the positive manifolds of level and change intercorrelations persisted after controlling for the extensive set of covariates described earlier. All levels and changes were simultaneously regressed onto the covariates and the residual intercorrelations estimated.

Table 7 reports the unique associations between the covariates and the level and change of cognitive abilities estimated by a multiple regression that included all covariates. A number of consistent patterns emerged with respect to levels of performance. First, even within this very narrow age cohort, older age was associated with lower performance across all four abilities. Second, both childhood intelligence and ultimate educational attainment uniquely predicted each of the four abilities. Childhood intelligence predicted each of the four abilities at nearly identical magnitudes (.240–.274), whereas educational attainment had noticeably stronger associations with crystallized ability and memory than with visuospatial ability and processing speed (.407 and .304 compared with .219 and .163, respectively). Third, being male was uniquely associated with lower performance across multiple domains. Fourth, higher forced expiratory volume and faster walk time were specifically related to processing speed and weakly with visuospatial ability, whereas stronger grip strength had more general associations with all abilities except for memory. Fifth, *APOE* genotype was significantly uniquely associated with visuospatial ability and processing speed, but not memory or crystallized ability, with presence of the ε4 allele conferring risk for lower performance. Finally, being a smoker was significantly associated with lower visuospatial ability and processing speed and marginally associated with lower memory performance. Cardiovascular disease, hypertension status, and diabetes diagnosis tended to not be uniquely associated with performance beyond the other predictors in the regression model at statistically significant levels. Minor exceptions were small associations between cardiovascular disease and slower processing speed and between diabetes diagnosis and crystallized ability performance.[Table-anchor tbl7]

Relations between the covariates and changes in performance tended to be smaller in magnitude, and fewer were statistically significant. For memory and crystallized ability, being older was actually associated with significantly less decline, a somewhat unexpected finding that may be attributable to idiosyncrasies associated with the very narrow age range of our sample. Childhood intelligence was not associated with change in any of the abilities except for processing speed, for which the effect was negative, indicating that higher childhood intelligence was associated with faster aging-related declines in processing speed. Men appeared to experience somewhat steeper declines than women, but the association between sex and change was only significant for crystallized ability. Faster walk time was marginally associated with less memory decline and processing speed decline. Grip strength was marginally associated with crystallized ability change, in the direction of less decline for those with stronger grip strength. *APOE* genotype was marginally associated with memory change, with carriers of the ε4 allele showing greater decline. Cardiovascular disease status, hypertension status, being a smoker, and diabetes diagnosis were not uniquely associated with decline beyond the other predictors in the model.

Table 8 reports correlations among level factors, among latent difference scores, and between levels and latent difference scores after controlling for all of the above-described covariates. Also provided are factor loadings from an alternative model in which, rather than allowing for factor intercorrelations, a higher order general factor of levels and a separate higher order general factor of changes were fit, again controlling for all covariates. It can be seen that the positive manifolds of both level and change intercorrelations persisted at nearly full strength. Level correlations were attenuated by an average of .126 correlation units (a 20% reduction), and change correlations were attenuated by an average of .008 correlation units (a 2% reduction). The average proportions of covariate-independent variance accounted for by the general factors of levels and change were 52% and 43%, respectively, compared with 64% and 48% in models that did not control for covariates. Alternatively put, the proportion of variance in levels accounted for by the general level factor was attenuated by only 18% (i.e., [64% − 52%]/64%) after controlling for the covariates, and the proportion of variance in the changes accounted for by the general change factor was attenuated by only 10% (i.e., [48% − 43%]/48%) after controlling for the covariates. These results indicate that the general factor of cognitive change is not simply an epiphenomenon of changes in different cognitive abilities being similarly related to the common set of risk factors examined. Again, differences persisted in terms of the variance accounted for by the general factor for crystallized ability compared with the other abilities. For initial levels, the general factor accounted for 26% of the variance in crystallized ability, compared with an average of 61% for the other three domains. The pattern was similar, but smaller, for amount of variance in change accounted for by the general factor (33% for crystallized ability change compared with 46% on average for the other three domains).[Table-anchor tbl8]

## Discussion

In a population-based, narrow-age cohort sample of 70-year-old adults, we found moderate to strong correlations between 3-year longitudinal changes in visuospatial ability, processing speed, memory, and crystallized ability. A common factor fit to the longitudinal change factors accounted for nearly 50% of the variation in longitudinal changes. Importantly, this pattern was robust to controls for a host of variables implicated in previous research as possible risk or protective factors in cognitive aging, including educational attainment, childhood intelligence, physical function, *APOE* genotype, smoking status, diagnosis of hypertension, diagnosis of cardiovascular disease, and diagnosis of diabetes (Plassman et al., 2010). These results suggest that concomitant changes in multiple domains of cognitive function are a core feature of cognitive aging.

The longitudinal interval for the current study was fairly short compared with typical longitudinal studies of cognitive aging. Had we reported null results, it would have been sensible to question whether failures to detect change interrelations derived from the relatively short time lag, over which there was limited opportunity for substantial heterogeneity in changes to accrue. However, given that we detected change interrelations that were not only statistically significant but moderate to large in magnitude, the relatively short time lag of our study can be considered a strength that speaks to the robustness of the phenomenon uncovered. Moreover, that our study was based on a narrow-age cohort of 70-year-olds followed over time ensured that the intercorrelations between rates of change were driven by the passage of time, rather than age-based heterogeneity. Although a potential limitation of narrow-age cohort studies is that results may not generalize to individuals from other cohorts, the results of the prior studies indexed in Table 1 indicate that shared variance in cognitive change is likely to be a rule rather than an exception in normal cognitive aging.

Our success at detecting systematic correlations among rates of change can also be attributable to the implementation of latent difference score models. Latent difference score models, along with growth curve models, belong to a class of quantitative models that separate systematic change from measurement error. Our study adds to the growing body of research, indexed in Table 1, that has implemented multivariate versions of this powerful class of models to examine interrelations among changes in multiple cognitive variables. Without implementing such models (i.e., if observed difference scores were to be implemented), the ratio of true change to error (what might be termed a signal-to-noise ratio) is typically so vast that correlations are obscured to the point of being undetectable. In such instances, more prolonged longitudinal intervals would be necessary for systematic changes to accumulate to the degree that correlations become detectable. Indeed, based on previous simple difference score analyses of single cognitive tests and other single variables in these LBC1936 data, Johnson et al. (2012) concluded that “two waves of longitudinal data were not sufficient to assess meaningful patterns of ageing” (p. 312).

Although we view the latent difference score approach implemented in the current study and growth curve approaches implemented in previous articles as being similar in their capabilities to separate systematic change variance from error variance, there are some important distinctions between these two classes of models that one should keep in mind when interpreting the current results. The distinction largely surrounds the meaning of the term *error* (see Crocker & Algina, 1986). Under a classical test theory perspective, measurement error reflects a failure of the measurement instrument to perfectly capture the true score of the individual during the period of cognitive testing. Under more dynamic longitudinal perspectives, state error reflects a fairly short-term, reversible, deviation from an individual’s more stable trait-level ability. Importantly, a measurement error-free index of a sample of individuals’ true scores during a circumscribed testing period represents a mixture of variation attributable to those individuals’ trait levels and their momentary states (see Nesselroade, 1991). Indeed, when the research question is focused on trait change, as it was here, latent difference score approaches may undercorrect for error by eliminating measurement error but not state error, and growth curve approaches may overcorrect for error by eliminating measurement error, state error, and (often nonlinear) developmental change that does not conform to the (often linear) function chosen. The implications of these nuanced differences for examining correlated aging-related changes are complex. On the one hand, if variation in state error is small relative to variation in trait change, then latent difference score models and properly specified growth curve models should largely capture the same components of change and produce very similar results with respect to the magnitude of change intercorrelations. On the other hand, if variation in state error is nontrivial, latent difference score approaches may either underestimate change intercorrelations (if states are uncorrelated across abilities, i.e., if individuals’ “good days” for processing speed are not particularly likely to coincide with their “good days” for memory) or overestimate change intercorrelations (if states are correlated across abilities, i.e., if individuals’ ‘“good days” for processing speed are likely to coincide with their “good days” for memory). Although more work will be necessary to directly compare growth curve and latent difference score approaches to cognitive aging in the same data set, results from different approaches have largely been consistent with one another. For instance, Tucker-Drob (2011a) found similar solutions for an exploratory factor analysis of latent difference scores of cognitive variables and a confirmatory factor analysis of growth curve slopes of the same variables. Moreover, Hertzog, Dixon, Hultsch, and MacDonald (2003) and Zimprich and Martin (2002), whose studies were based on latent difference scores, produced estimates of shared variance that were very similar to estimates produced by the other articles summarized in Table 1, which were based on growth curve approaches.

Whereas a strength of our study is that we were able to examine the key phenomenon of interest both before and after accounting for a host of covariates, it is important to keep in mind that the covariates were much more consistent in accounting for individual differences in levels of cognitive performance than individual differences in cognitive change. This observation is not unique to the current study. For instance, our results agree with many previous rigorous studies (e.g., Tucker-Drob, Johnson, & Jones, 2009; Van Dijk, Van Gerven, Van Boxtel, Van der Elst, & Jolles, 2008; Wilson, Barnes, Mendes de Leon, & Evans, 2009; Zahodne et al., 2011) in indicating that educational attainment was related to levels of cognitive abilities but was unrelated to rates of longitudinal cognitive change, despite theoretical speculations to the contrary (e.g., Stern, 2002). Identifying systematic correlates of aging-related cognitive changes has been an ongoing challenge in the cognitive aging literature (Salthouse, 2006; although see Hertzog, Kramer, Wilson, & Lindenberger, 2008, for a more optimistic review of the literature). Future research should continue to examine correlates of longitudinal changes rather than simple levels of performance. As is the case that concurrent (cross-sectional) correlations among cognitive tasks are ambiguous with respect to the codependency in late life, concurrent correlations among risk factors and levels of performance are ambiguous with respect to direction and timing of causation (for empirical examples and solutions, see Corley et al., 2011, and Luciano, Marioni, Gow, Starr, & Deary, 2009).

We consistently found that longitudinal changes in crystallized ability were less coupled with general change found for other abilities. In fact, latent difference scores for this domain had means that were closest to zero and displayed the least amount variance of all the domains examined. Ghisletta, Rabbitt, Lunn, and Lindenberger (2012) and Lindenberger and Ghisletta (2009) have reported similar results. These results are together consistent with theoretical propositions that the aging of crystallized abilities, to a considerable extent, represents a mechanistically distinct process from that of other cognitive domains. For instance, Baltes and Staudinger (1993) described this type of divergent trend as evidence for somewhat distinct processes of “the ‘biological’ cognitive *mechanics* and the ‘cultural’ cognitive *pragmatics*” (p. 75). On the whole, however, that longitudinal changes in all four domains were significantly correlated with one another suggests the operation of a common domain-general dimension of cognitive aging.

Our finding that a single broad dimension could statistically account for sizable proportions of variation in aging-related changes in more specific cognitive abilities does not undermine the clear evidence that cognitive aging is a highly multidetermined phenomenon. Although there may be many individual causal factors contributing to variation in cognitive aging, our results indicate that there is a tendency either for these factors to themselves be correlated and/or for their effects to operate on a broad range of cognitive abilities. Plomin and Spinath (2002) provide a similar discussion with respect to the interpretation of the *g* factor of individual differences in levels of cognitive abilities.

Deary (2000) described the *g* factor of individual differences in intelligence to be “arguably the most replicated result in all psychology” (p. 6). The current findings add to a very consistent body of work, indexed in Table 1, indicating a perhaps similarly robust factor of cognitive change in adulthood. Moreover, an investigation of developmental change in early childhood also produced strong evidence for a general factor of longitudinal change (Rhemtulla & Tucker-Drob, 2011). A question that therefore arises is whether the common factor of levels and the common factor of changes reflect the same underlying phenomenon. Juan-Espinosa et al. (2002), for instance, have posited that the structure of individual differences in cognition is inherent to the human system, arguing that “basic structure does not change at all, although, like the human bones, the cognitive abilities grow up and decline at different periods of life” (p. 407). If the human cognitive system is indeed structured along invariant intrinsic dimensions, then it may be the case that individual differences in longitudinal cognitive changes are necessarily structured to change in concert. Alternative views of the mechanisms underlying the *g* factor, however, make no such presumption of basic immutable structure. For instance, van der Maas et al. (2006) argued that the structure of cognitive abilities is an emergent property of mutually reinforcing dependencies between different abilities (see Dickens, 2007, for a similar perspective, and Tucker-Drob, 2009, for further discussion of multiple perspectives on the etiology of factor structure). If these dependencies change or subside in old age, then the same positive manifold as is typically observed for levels might not be expected to necessarily occur for rates of change. However, if the dependencies between different abilities are maintained, then a positive manifold of changes would be evident in spite of not having been caused by a single underlying factor or an otherwise immutable structure.

In conclusion, our results add to the growing body of literature indicating strong codependencies between individual differences in rates of longitudinal cognitive changes. We demonstrate that a positive manifold of cognitive change intercorrelations can be detected over a relatively short period of time (3 years) in a narrow-age cohort and persists even after controlling for a variety of covariates. Future work will be needed to understand the mechanisms that give rise to these statistical codependencies.

## Figures and Tables

**Table 1 tbl1:** Findings From Past Studies Reporting Relations Among Rates of Change in Two or More Cognitive Variables

Study	*n* (for occasions ≥ 2)	Age range (years)	Maximum time span (years)	Maximum assessments	Variable	Shared variance (%)
Anstey, Hofer, & Luszcz (2003)	1,423	65–85 +	8	3	Memory factor, speed factor	62
Ferrer Salthouse, McArdle, & Stewart (2005) ALEND data	717	40–70	4	4	Processing speed composite, verbal memory composite	63
Ferrer Salthouse, McArdle, & Stewart (2005) NGCS data	381	30–80	10+	3	Processing speed composite, verbal memory composite	58
Hertzog et al. (2003)	303	61–91	6	2	Working memory factor, reaction time factor, processing speed factor, induction factor, fact recall factor, word recall factor, story recall factor, vocabulary factor	41
Ghisletta et al. (2012)	4,458	43–93	20	7	Multiple individual tests of fluid intelligence, crystallized intelligence, perceptual speed, and memory	66
Lindenberger & Ghisletta (2009)	361	70–103	13	6	Digit letter, identical pictures; paired associates; memory for text, categories	60
Sliwinski & Buschke (2004)	244	65+	6.5+	6	Memory variable, speed variable	33
				4	Speed variable, fluency variable	33
					Fluency variable, memory variable	16
Sliwinski, Hofer, & Hall (2003)	467	73–92	7	12	Fluency variable, memory variable	61 [42]
					Memory variable, speed variable	56 [48]
					Fluency variable, speed variable	56 [34]
Tucker-Drob (2011a)	1,281	18–95	7	2	Abstract reasoning, spatial visualization, episodic memory, processing speed	63
Tucker-Drob (2011b)	639	65–94	6	6	Reasoning composite, processing speed composite, episodic memory composite, everyday problem-solving variable, observed tasks of daily living variable, timed instrumental activities of daily living variable	66
Tucker-Drob, Briley, Starr, & Deary (Current Report)	866	67–71	4.8	2	Visuospatial ability, processing speed, memory, crystallized ability	48
Tucker-Drob, Reynolds, Finkel, & Pedersen (2014)	747	50–96	16	5	Verbal ability composite, spatial ability composite, memory composite, processing speed composite	78
Wilson et al. (2002)	596	65–90+	6	6	Story retention, word retention, word generation, word knowledge, working memory, perceptual speed, visuospatial ability	61.8
Zimprich & Martin (2002)	417	62–64	4	2	Fluid intelligence factor, processing speed factor	53
*Note*. In constructing this table, if we identified multiple relevant papers based on data from the same sample, we included results from only one paper (typically the paper with the largest sample size, greatest number of occasions, and/or the largest number of cognitive abilities). Shared variance reflects communalities for factor models when more than two indices are listed and bivariate correlations when only two indices are listed. It can be shown that, in the bivariate case, correlations are equivalent to factor communalities. Terms in brackets are after probable preclinical cases of dementia were excluded. ALEND = Age, Lead Exposure, and Neurobehavioral Decline; NGCS = National Growth and Change Study.						

**Table 2 tbl2:** Descriptive Statistics of Study Cognitive Variables

Cognitive indicator	Reliability	Mean		*SD*															
		Time 1	Time 2	Time 1	Time 2	1	2	3	4	5	6	7	8	9	10	11	12	13	14
Visuospatial																			
1. Matrix Reasoning	.91	13.493	12.896	5.127	4.997	—	.539	.293	.329	.389	.387	.285	−.261	.331	.305	.351	.401	.406	.221
2. Block Design	.83	33.766	33.069	10.317	10.184	.571	—	.357	.416	.491	.407	.323	−.328	.266	.274	.294	.365	.380	.243
3. Spatial Span Forward	.75	7.677	7.041	1.643	1.664	.242	.249	—	.429	.308	.266	.245	−.293	.184	.158	.252	.153	.167	.154
4. Spatial Span Backward	.75	7.041	6.995	1.741	1.615	.386	.416	.397	—	.374	.303	.280	−.311	.228	.199	.271	.173	.178	.180
Processing Speed																			
5. Symbol Search	.79	24.700	24.235	6.382	6.234	.448	.483	.289	.390	—	.637	.390	−.513	.296	.240	.331	.374	.388	.338
6. Digit Symbol	.86	56.601	55.538	12.935	12.494	.365	.395	.212	.297	.618	—	.398	−.560	.333	.267	.330	.417	.413	.385
7. Inspection Time	≥.59	111.975	110.503	11.034	11.971	.219	.278	.230	.233	.329	.308	—	−.405	.217	.206	.212	.249	.242	.268
8. Choice RT Mean	.92	.642	.655	.084	.089	−.268	−.319	−.279	−.323	−.480	−.515	−.368	—	−.282	−.244	−.223	−.254	−.243	−.309
Memory																			
9. Logical Memory	.81	44.058	45.021	10.438	10.571	.323	.270	.146	.244	.324	.306	.072	−.217	—	.521	.322	.434	.411	.249
10. Verbal PA	.94	20.103	20.229	7.460	7.869	.306	.272	.113	.160	.211	.246	.093	−.171	.454	—	.287	.375	.370	.316
11. Digits Backwards	.86	7.735	7.726	2.262	2.284	.401	.336	.254	.285	.342	.302	.177	−.252	.301	.260	—	.393	.409	.321
Crystallized																			
12. NART	.90	34.481	34.036	8.149	8.221	.452	.404	.117	.245	.397	.398	.129	−.249	.448	.356	.433	—	.907	.455
13. WTAR	.90	41.021	40.770	7.170	7.043	.441	.386	.151	.254	.410	.378	.151	−.237	.447	.358	.438	.894	—	.456
14. Verbal Fluency	.88	42.401	42.770	12.535	12.874	.284	.258	.130	.158	.368	.358	.171	−.239	.231	.244	.311	.467	.472	—
*Note*. RT = reaction time; PA = Paired Associates; NART = National Adult Reading Test; WTAR = Wechsler Test of Adult Reading. Correlations among Time 1 cognitive variables are presented below the diagonal, and correlations among Time 2 cognitive variables are presented above the diagonal. For Matrix Reasoning, Block Design, Spatial Span, Logical Memory, Verbal PA, and Digits Backwards, split-half reliabilities were derived from Wechsler (1997). For Spatial Span Forward, Spatial Span Backward, and Digits Backwards, reliabilities were not available for subscores; therefore, reliabilities from Spatial Span Total and Digit Span Total are used. Reliabilities for Digit Symbol and Symbol Search are test–retest reliabilities derived from Wechsler (1997). For WTAR, split-half reliability was derived from Wechsler (2001). For NART, split-half reliability was derived from Crawford, Stewart, Garthwaite, Parker, and Besson (1988). Reliability of Verbal Fluency is a Cronbach’s alpha estimate from the baseline wave of the Lothian Birth Cohort 1936. Reliability for Choice Reaction Time is a test–retest correlation up to 1 day, derived from Deary and Der (2005). Internal consistency/short-term test–retest stability for Inspection Time was unavailable. We therefore indicate that the reliability of Inspection Time is greater than or equal to its 3-year test–retest stability, which is reported in Johnson et al. (2012).																			

**Table 3 tbl3:** Model Fit Comparison for Tests of Measurement Invariance

Invariance	χ^2^	*df*	*p*	RMSEA	AIC	BIC	Δχ^2^	*p*
Configural	893.534	308	<.000	.042	141976.274	142605.509		
Weak factorial	930.548	318	<.000	.042	141993.288	142572.584	37.014	<.000
Strong factorial	961.437	328	<.000	.042	142004.177	142533.534	30.889	.001
Strict factorial	991.426	342	<.000	.042	142006.166	142465.608	29.989	.008
*Note*. RMSEA = root mean square error of approximation; AIC = Akaike information criterion; BIC = Bayesian information criterion. All Δχ^2^ tests are based on the model immediately preceding the specified model.								

**Table 4 tbl4:** Parameter Estimates for Tests of Measurement Invariance

Cognitive indicator	Baseline model: configural invariance						Final model: strict factorial invariance			
	λ		υ		σ_u_^2^		λ	λ_(std)_	υ	σ_u_^2^
	Time 1	Time 2	Time 1	Time 2	Time 1	Time 2	=		=	=
Visuospatial										
Matrix Reasoning	3.675	3.675	13.490	13.490	12.711	13.281	3.630	0.710	13.407	12.936
Block Design	7.691	8.136	33.768	34.367	47.070	45.793	7.725	0.749	33.831	46.701
Spatial Span Forward	0.680	0.894	7.677	7.720	2.241	2.081	0.750	0.452	7.673	2.183
Spatial Span Backward	1.010	0.983	7.041	7.152	2.016	1.766	0.982	0.579	7.066	1.909
Processing speed										
Symbol Search	5.105	5.105	24.698	24.698	14.679	14.260	4.996	0.795	24.786	14.535
Digit Symbol	9.841	10.160	56.600	56.470	69.765	57.636	9.751	0.771	56.706	64.759
Inspection Time	4.871	6.392	111.980	111.054	98.117	103.947	5.442	0.476	111.682	101.010
Choice RT mean	−0.055	−0.062	0.642	0.649	0.004	0.005	−0.057	−0.647	0.644	0.005
Memory										
Logical Memory	6.560	6.560	44.057	44.057	66.809	58.206	6.739	0.647	44.253	63.053
Verbal PA	4.099	4.417	20.120	19.597	39.110	37.083	4.324	0.572	20.051	38.396
Digits Backwards	1.275	1.078	7.735	7.574	3.399	3.677	1.208	0.542	7.693	3.511
Crystallized										
NART	7.708	7.708	34.481	34.481	6.947	5.886	7.765	0.950	34.455	6.507
WTAR	6.776	6.574	41.020	41.077	5.513	5.007	6.741	0.946	41.020	5.314
Verbal Fluency	6.311	6.192	42.404	43.195	118.043	126.181	6.273	0.494	42.705	121.624
*Note*. RT = reaction time; PA = Paired Associates; NART = National Adult Reading Test; WTAR = Wechsler Test of Adult Reading. “=” indicates that the parameter was constrained to be equal across time. All loadings, intercepts, and residual variances are significant at *p* < .001 in each model. All parameters are unstandardized except for column labeled “λ_(std)_,” which presents standardized loadings for the final model. The unstandardized loading and intercept of the first indicator of each factor were constrained to be equal across time.										

**Table 5 tbl5:** Means and Variances of Latent Difference Scores

Factor	Slope mean	Δχ^2^	*p*	Slope variance	Δχ^2^	*p*
1. Visuospatial	−.112 (.023)	24.580	<.000	.048 (.025)	3.639	.056
2. Processing Speed	−.137 (.019)	52.406	<.000	.073 (.017)	22.633	<.000
3. Memory	.072 (.030)	5.747	.017	.257 (.048)	37.383	<.000
4. Crystallized	−.048 (.010)	24.871	<.000	.022 (.005)	25.141	<.000
*Note*. Standard errors are reported in parentheses. Nested χ^2^ comparisons were used to test the significance of each parameter. This entailed comparing the fit of a model in which the parameter was freely estimated with a model with the parameter constrained to zero. The baseline model has a χ^2^ value of 991.426 and 342 *df*. The comparison models differed from the baseline model by 1 *df* and a χ^2^ value by the amount listed in the table. Because they were calculated from a conventional χ^2^ distribution, the *p* values reported above for the slopes are likely to be conservative, as a number of methodological articles have indicated that, to account for the fact that variances are bounded at zero, the conventionally calculated *p* value should be divided by 2 (Stoel, Garre, Dolan, & van den Wittenboer, 2006; Dominicus, Skrondal, Gjessing, Pedersen, & Palmgren, 2006).						

**Table 6 tbl6:** Correlations Among Levels and Changes

Factor	Parameter estimates and *SE*s from full level and change correlation matrix				Parameter estimates and *SE*s from general level and change factor model
	Visuospatial	Processing Speed	Memory	Crystallized	Standardized loading on level factor
Level-Level					
Visuospatial	—				.849 (.023)***
Processing Speed	.751 (.023)***	—			.782 (.022)***
Memory	.705 (.034)***	.598 (.034)***	—		.866 (.031)***
Crystallized	.554 (.027)***	.503 (.027)***	.741 (.027)***	—	.704 (.023)***
	Δ Visuospatial	Δ Processing Speed	Δ Memory	Δ Crystallized	Standardized loading on change factor
Change-Change					
Δ Visuospatial	—				.834 (.271)**
Δ Processing Speed	.451 (.235)^†^	—			.735 (.148)***
Δ Memory	.595 (.232)*	.475 (.128)***	—		.614 (.123)***
Δ Crystallized	.497 (.229)*	.385 (.135)**	.362 (.119)*	—	.547 (.129)***
	Δ Visuospatial	Δ Processing Speed	Δ Memory	Δ Crystallized	
Level-Change					
Visuospatial	−.236 (.110)*	.055 (.080)	.027 (.069)	−.038 (.075)	
Processing Speed	−.190 (.123)	−.060 (.078)	.054 (.067)	.037 (.072)	
Memory	−.388 (.160)*	−.021 (.089)	−.094 (.078)	−.054 (.084)	
Crystallized	−.320 (.128)*	.000 (.071)	−.038 (.061)	−.073 (.066)	
^†^ *p* = .06. * *p* < .05. ** *p* < .01. *** *p* < .001.					

**Table 7 tbl7:** Demographic, Physical Health, and Medical Risk Predictors of Levels and Change

Predictor	Standardized multiple regression coefficients			
	Visuospatial	Processing Speed	Memory	Crystallized
Level				
Age (baseline)	−.141 (.038)***	−.190 (.037)***	−.248 (.044)***	−.122 (.031)***
Time lag	.015 (.041)	−.027 (.040)	−.036 (.048)	.012 (.033)
Age 11 intelligence	.260 (.032)***	.240 (.031)***	.247 (.037)***	.274 (.026)***
Educational attainment	.219 (.032)***	.163 (.031)***	.304 (.037)***	.407 (.025)***
Male^a^	−.020 (.103)	−.516 (.099)***	−.385 (.119)**	−.349 (.083)***
Forced expiratory volume (baseline)	.080 (.043)^†^	.141 (.042)**	−.006 (.050)	.041 (.035)
–1*Log 6-m walk time (baseline)	.075 (.033)*	.133 (.033)***	.025 (.039)	.052 (.027)^†^
Grip strength (baseline)	.225 (.053)***	.151 (.052)**	.082 (.062)	.118 (.043)**
*APOE* genotype (ε4 allele)^a^	−.216 (.067)**	−.202 (.065)**	−.079 (.078)	−.011 (.055)
Smoking status (baseline)^a^	−.267 (.098)**	−.260 (.095)**	−.191 (.113)^†^	−.012 (.080)
Cardiovascular disease status (baseline)^a^	−.084 (.072)	−.191 (.070)**	.056 (.083)	−.005 (.059)
Hypertension status (baseline)^a^	−.047 (.064)	−.046 (.062)	−.006 (.074)	−.065 (.052)
Diabetes diagnosis (baseline)^a^	−.154 (.093)^†^	−.142 (.091)	−.145 (.108)	−.159 (.076)*
	Δ Visuospatial	Δ Processing Speed	Δ Memory	Δ Crystallized
Changes				
Age (baseline)	.194 (.139)	.103 (.083)	.230 (.071)**	.268 (.080)**
Time lag	−.077 (.131)	−.143 (.083)^†^	−.050 (.071)	−.116 (.077)
Age 11 intelligence	−.189 (.122)	−.171 (.073)*	.022 (.062)	.003 (.068)
Educational attainment	−.072 (.115)	−.001 (.073)	−.052 (.062)	.039 (.068)
Male^a^	−.108 (.363)	−.375 (.234)	−.236 (.200)	−.515 (.220)*
Forced expiratory volume (baseline)	.090 (.152)	.000 (.096)	.033 (.082)	.048 (.090)
–1*Log 6-m walk time (baseline)	.142 (.125)	.134 (.077)^†^	.126 (.066)^†^	−.094 (.073)
Grip strength (baseline)	.069 (.188)	.188 (.120)	.161 (.103)	.200 (.113)^†^
*APOE* genotype (ε4 allele)^a^	−.073 (.236)	−.153 (.151)	−.250 (.129)^†^	.223 (.142)
Smoking status (baseline)^a^	−.218 (.380)	−.268 (.242)	.210 (.208)	−.223 (.229)
Cardiovascular disease status (baseline)^a^	−.322 (.267)	.003 (.165)	−.022 (.141)	−.047 (.155)
Hypertension status (baseline)^a^	.017 (.227)	.060 (.146)	.015 (.125)	.157 (.137)
Diabetes diagnosis (baseline)^a^	.190 (.327)	−.115 (.206)	−.074 (.176)	.092 (.194)
*Note*. Standardized multiple regression coefficients represent the unique effect of each predictor. Standard errors are reported in parentheses.				
^a^ Predictor was dichotomous. Parameter estimates for dichotomous predictors were standardized with respect to the outcome (but not the predictor) such that they can be interpreted as Cohen’s *d* effect sizes. All other parameters estimates were standardized with respect to both the predictor and the outcome.				
^†^ *p* < .10. * *p* < .05. ** *p* < .01. *** *p* < .001.				

**Table 8 tbl8:** Correlations Among Levels and Changes Controlling for Demographic, Physical Health, and Medical Risk Factors

Factor	Parameter estimates and *SE*s from full level and change correlation matrix				Parameter estimates and *SE*s from general level and change factor model
	Visuospatial	Processing Speed	Memory	Crystallized	Standardized loading on level factor
Level-Level					
Visuospatial	—				.849 (.034)***
Processing Speed	.678 (.031)***	—			.705 (.031)***
Memory	.665 (.044)***	.463 (.044)***	—		.785 (.046)***
Crystallized	.377 (.035)***	.296 (.034)***	.615 (.036)***	—	.509 (.034)***
	Δ Visuospatial	Δ Processing Speed	Δ Memory	Δ Crystallized	Standardized loading on change factor
Change-Change					
Δ Visuospatial	—				.710 (.306)*
Δ Processing Speed	.425 (.274)	—			.742 (.171)***
Δ Memory	.599 (.277)*	.437 (.137)**	—		.582 (.135)***
Δ Crystallized	.537 (.286)^†^	.371 (.152)*	.347 (.131)**	—	.577 (.149)***
	Δ Visuospatial	Δ Processing Speed	Δ Memory	Δ Crystallized	
Level-Change					
Visuospatial	−.214 (.127)	.116 (.088)	.025 (.075)	.032 (.085)	
Processing Speed	−.161 (.140)	−.049 (.085)	.072 (.071)	.020 (.080)	
Memory	−.317 (.185)	.060 (.100)	−.060 (.089)	−.023 (.097)	
Crystallized	−.259 (.141)	.086 (.075)	−.022 (.064)	−.073 (.072)	
^†^ *p* = .06. * *p* < .05. ** *p* < .01. *** *p* < .001.					

## References

[c1] Anstey K. J., Hofer S. M., & Luszcz M. A. (2003). A latent growth curve analysis of late-life sensory and cognitive function over 8 years: Evidence for specific and common factors underlying change. Psychology and Aging, 18, 714–726. doi:10.1037/0882-7974.18.4.714 14692859

[c2] Baltes P. B., Nesselroade J. R., & Cornelius S. W. (1978). Multivariate antecedents of structural change in development: A simulation of cumulative environmental patterns. Multivariate Behavioral Research, 13, 127–152. doi:10.1207/s15327906mbr1302_1 26794014

[c3] Baltes P. B., & Staudinger U. M. (1993). The search for a psychology of wisdom. Current Directions in Psychological Science, 2, 75–80. doi:10.1111/1467-8721.ep10770914

[c4] Corley J., Jia X., Brett C. E., Gow A. J., Starr J. M., Kyle J. A. M., . . . Deary I. J. (2011). Alcohol intake and cognitive abilities in old age: The Lothian Birth Cohort 1936 study. Neuropsychology, 25, 166–175. doi:10.1037/a0021571 21381824

[c5] Crawford J. R., Stewart L. E., Garthwaite P. H., Parker D. M., & Besson J. A. O. (1988). The relationship between demographic variables and NART performance in normal subjects. British Journal of Clinical Psychology, 27, 181–182. doi:10.1111/j.2044-8260.1988.tb00770.x 3395744

[c6] Crocker L., & Algina J. (1986). Introductions to classical and modern test theory. New York, NY: Holt, Rinehart & Winston

[c7] Cronbach L. J., & Furby L. (1970). How we should measure “change”: Or should we? Psychological Bulletin, 74, 68–80. doi:10.1037/h0029382

[c8] Deary I. J. (2000). Looking down on human intelligence: From psychometrics to the brain. Oxford, UK: Oxford University Press. doi:10.1093/acprof:oso/9780198524175.001.0001

[c9] Deary I. J., & Der G. (2005). Reaction time parameters, intelligence, ageing, and death: The West of Scotland Twenty-07 study In Measuring the mind: Speed, control, and age (pp. 115–136). Oxford, UK: Oxford University Press. doi:10.1093/acprof:oso/9780198566427.003.0005

[c10] Deary I. J., Der G., & Ford G. (2001). Reaction times and intelligence differences: A population-based cohort study. Intelligence, 29, 389–399. doi:10.1016/S0160-2896(01)00062-9

[c11] Deary I. J., Gow A. J., Pattie A., & Starr J. M. (2012). Cohort profile: The Lothian birth cohorts of 1921 and 1936. International Journal of Epidemiology, 41, 1576–1584. doi:10.1093/ije/dyr197 22253310

[c12] Deary I. J., Gow A. J., Taylor M. D., Corley J., Brett C., Wilson V., . . . Starr J. M. (2007). The Lothian Birth Cohort 1936: A study to examine influences on cognitive ageing from age 11 to age 70 and beyond. BMC Geriatrics, 7, 28. doi:10.1186/1471-2318-7-28 18053258 PMC2222601

[c13] Deary I. J., Simonotto E., Meyer M., Marshall A., Marshall I., Goddard N., & Wardlaw J. M. (2004). The functional anatomy of inspection time: An event-related fMRI study. NeuroImage, 22, 1466–1479. doi:10.1016/j.neuroimage.2004.03.047 15275904

[c14] Deary I. J., Whalley L. J., & Starr J. M. (2009). A lifetime of intelligence. Washington, DC: American Psychological Association

[c15] Deary I. J., Whiteman M. C., Pattie A., Starr J. M., Hayward C., Wright A. F., . . . Whalley L. J. (2004). Apolipoprotein e gene variability and cognitive functions at age 79: A follow-up of the Scottish mental survey of 1932. Psychology and Aging, 19, 367–371. doi:10.1037/0882-7974.19.2.367 15222832

[c16] Dickens W. T. (2007, 5 3). What is g? Retrieved from http://www.brookings.edu/research/papers/2007/05/03education-dickens

[c17] Dominicus A., Skrondal A., Gjessing H. K., Pedersen N. L., & Palmgren J. (2006). Likelihood ratio tests in behavioral genetics: Problems and solutions. Behavior Genetics, 36, 331–340. doi:10.1007/s10519-005-9034-7 16474914

[c18] Ferrer E., Salthouse T. A., McArdle J. J., & Stewart W. F. (2005). Multivariate modeling of age and retest in longitudinal studies of cognitive abilities. Psychology and Aging, 20, 412–422. doi:10.1037/0882-7974.20.3.412 16248701 PMC3838960

[c20] Ghisletta P., Rabbitt P., Lunn M., & Lindenberger U. (2012). Two thirds of age-based changes in fluid and crystallized intelligence, perceptual speed, and memory in adulthood are shared. Intelligence, 40, 260–268. doi:10.1016/j.intell.2012.02.008

[c21] Harris S. E., & Deary I. J. (2011). The genetics of cognitive ability and cognitive ageing in healthy older people. Trends in Cognitive Sciences, 15, 388–394. doi:10.1016/j.tics.2011.07.004 21840749

[c22] Hertzog C., Dixon R. A., Hultsch D. F., & MacDonald S. W. S. (2003). Latent change models of adult cognition: Are changes in processing speed and working memory associated with changes in episodic memory? Psychology and Aging, 18, 755–769. doi:10.1037/0882-7974.18.4.755 14692862

[c23] Hertzog C., Kramer A. F., Wilson R. S., & Lindenberger U. (2008). Enrichment effects on adult cognitive development: Can the functional capacity of older adults be preserved and enhanced? Psychological Science in the Public Interest, 9, 1–65. doi:10.1111/j.1539-6053.2009.01034.x 26162004

[c24] Hofer S. M., Flaherty B. P., & Hoffman L. (2006). Cross-sectional analysis of time-dependent data: Mean-induced association in age-heterogeneous samples and an alternative method based on sequential narrow age-cohort samples. Multivariate Behavioral Research, 41, 165–187. doi:10.1207/s15327906mbr4102_4 26782909

[c25] Hofer S. M., & Sliwinski M. J. (2001). Understanding ageing. Gerontology, 47, 341–352. doi:10.1159/000052825 11721149

[c26] Johnson W., & Deary I. J. (2011). Placing inspection time, reaction time, and perceptual speed in the broader context of cognitive ability: The VPR model in the Lothian Birth Cohort 1936. Intelligence, 39, 405–417. doi:10.1016/j.intell.2011.07.003

[c27] Johnson W., Gow A., Corley J., Redmond P., Henderson R., Murray C., . . . Deary I. (2012). Can we spot deleterious ageing in two waves of data? The Lothian Birth Cohort 1936 from ages 70 to 73. Longitudinal and Life Course Studies, 3

[c28] Juan-Espinosa M., García L. F., Escorial S., Rebollo I., Colom R., & Abad F. J. (2002). Age dedifferentiation hypothesis: Evidence from the WAIS III. Intelligence, 30, 395–408. doi:10.1016/S0160-2896(02)00092-2

[c29] Lezak M. (2004). Neuropsychological testing. Oxford, UK: Oxford University Press

[c30] Lindenberger U., & Ghisletta P. (2009). Cognitive and sensory declines in old age: Gauging the evidence for a common cause. Psychology and Aging, 24, 1–16. doi:10.1037/a0014986 19290733

[c31] Lindenberger U., Von Oertzen T., Ghisletta P., & Hertzog C. (2011). Cross-sectional age variance extraction: What’s change got to do with it? Psychology and Aging, 26, 34–47. doi:10.1037/a0020525 21417539

[c32] Luciano M., Marioni R., Gow A. J., Starr J. M., & Deary I. J. (2009). Reverse causation in the association between C-reactive protein and fibrinogen levels and cognitive abilities in an aging sample. Psychosomatic Medicine, 71, 404–409. doi:10.1097/PSY.0b013e3181a24fb9 19398500

[c33] McArdle J. J. (2009). Latent variable modeling of differences and changes with longitudinal data. Annual Review of Psychology, 60, 577–605. doi:10.1146/annurev.psych.60.110707.163612 18817479

[c34] McArdle J. J., & Nesselroade J. R. (2003). Growth curve analysis in contemporary psychological research. Handbook of Psychology, 3, 447–480. doi:10.1002/0471264385.wei0218

[c35] Muthén L. K., & Muthén B. O. (1998–2012). Mplus user’s guide (7th ed.). Los Angeles, CA: Author

[c36] Nelson H. E., & Willison J. (1991). National Adult Reading Test (NART). London, UK: NferNelson

[c37] Nesselroade J. R. (1991). The warp and the woof of the developmental fabric In Downs R. M., Liben L. S., & Palermo D. S. (Eds.), Visions of aesthetics, the environment and development: The legacy of Joachim Wohlwill (pp. 213–240). Hillsdale, NJ: Erlbaum

[c38] Plassman B. L., Williams J. W., Burke J. R., Holsinger T., & Benjamin S. (2010). Systematic review: Factors associated with risk for and possible prevention of cognitive decline in later life. Annals of Internal Medicine, 153, 182–193. doi:10.7326/0003-4819-153-3-201008030-00258 20547887

[c39] Plomin R., & Spinath F. M. (2002). Genetics and general cognitive ability (*g*). Trends in Cognitive Sciences, 6, 169–176. doi:10.1016/S1364-6613(00)01853-2 11912040

[c40] Rabbitt P. (1993). Does it all go together when it goes? The nineteenth Bartlett memorial lecture. Quarterly Journal of Experimental Psychology: Human Experimental Psychology, 46(A), 385–434. doi:10.1080/14640749308401055 8378549

[c41] Rhemtulla M., & Tucker-Drob E. M. (2011). Correlated longitudinal changes across linguistic, achievement, and psychomotor domains in early childhood: Evidence for a global dimension of development. Developmental Science, 14, 1245–1254. doi:10.1111/j.1467-7687.2011.01071.x 21884339 PMC5955711

[c42] Salthouse T. A. (2004). Localizing age-related individual differences in a hierarchical structure. Intelligence, 32, 541–561. doi:10.1016/j.intell.2004.07.003 PMC3866028 24357886

[c43] Salthouse T. A. (2006). Mental exercise and mental aging evaluating the validity of the “use it or lose it” hypothesis. Perspectives on Psychological Science, 1, 68–87. doi:10.1111/j.1745-6916.2006.00005.x 26151186

[c44] Salthouse T. A., Pink J. E., & Tucker-Drob E. M. (2008). Contextual analysis of fluid intelligence. Intelligence, 36, 464–486. doi:10.1016/j.intell.2007.10.003 19137074 PMC2615402

[c45] Salthouse T. A., & Tucker-Drob E. M. (2008). Implications of short-term retest effects for the interpretation of longitudinal change. Neuropsychology, 22, 800–811. doi:10.1037/a0013091 18999354 PMC2593909

[c46] Sliwinski M., & Buschke H. (2004). Modeling intraindividual cognitive change in aging adults: Results from the Einstein Aging Studies. Aging, Neuropsychology, and Cognition, 11, 196–211. doi:10.1080/13825580490511080

[c47] Sliwinski M., Hofer S. M., & Hall C. (2003). Correlated and coupled cognitive change in older adults with and without preclinical dementia. Psychology and Aging, 18, 672–683. doi:10.1037/0882-7974.18.4.672 14692856

[c48] Spearman C. (1904). “General intelligence,” objectively determined and measured. The American Journal of Psychology, 15, 201–293. doi:10.2307/1412107

[c49] Stern Y. (2002). What is cognitive reserve? Theory and research application of the reserve concept. Journal of the International Neuropsychological Society, 8, 448–460. doi:10.1017/S1355617702813248 11939702

[c50] Stoel R. D., Garre F. G., Dolan C., & van den Wittenboer G. (2006). On the likelihood ratio test in structural equation modeling when parameters are subject to boundary constraints. Psychological Methods, 11, 439–455. doi:10.1037/1082-989X.11.4.439 17154756

[c51] Tucker-Drob E. M. (2009). Differentiation of cognitive abilities across the life span. Developmental Psychology, 45, 1097–1118. doi:10.1037/a0015864 19586182 PMC2855504

[c52] Tucker-Drob E. M. (2011a). Global and domain-specific changes in cognition throughout adulthood. Developmental Psychology, 47, 331–343. doi:10.1037/a0021361 21244145 PMC5374863

[c53] Tucker-Drob E. M. (2011b). Neurocognitive functions and everyday functions change together in old age. Neuropsychology, 25, 368–377. doi:10.1037/a0022348 21417532 PMC3086995

[c54] Tucker-Drob E. M., Johnson K. E., & Jones R. N. (2009). The cognitive reserve hypothesis: A longitudinal examination of age-associated declines in reasoning and processing speed. Developmental Psychology, 45, 431–446. doi:10.1037/a0014012 19271829 PMC3230274

[c55] Tucker-Drob E. M., Reynolds C. A., Finkel D., & Pedersen N. L. (2014). Shared and unique genetic and environmental influences on aging-related changes in multiple cognitive abilities. Developmental Psychology, 50, 152–166. doi:10.1037/a0032468 23586942 PMC4135450

[c56] Tucker-Drob E. M., & Salthouse T. A. (2011). Individual differences in cognitive aging In Chamorro-Premuzic T., von Stumm S., & Furnham A. (Eds.), The Wiley-Blackwell handbook of individual differences (pp. 242–267). Malden, MA: Wiley-Blackwell

[c57] van der Maas H. L. J., Dolan C. V., Grasman R. P. P. P., Wicherts J. M., Huizenga H. M., & Raijmakers M. E. J. (2006). A dynamical model of general intelligence: The positive manifold of intelligence by mutualism. Psychological Review, 113, 842–861. doi:10.1037/0033-295X.113.4.842 17014305

[c58] Van Dijk K. R. A., Van Gerven P. W. M., Van Boxtel M. P. J., Van der Elst W., & Jolles J. (2008). No protective effects of education during normal cognitive aging: Results from the 6-year follow-up of the Maastricht Aging Study. Psychology and Aging, 23, 119–130. doi:10.1037/0882-7974.23.1.119 18361661

[c59] Wechsler D. (1997). WAIS–III/WMS–III technical manual. San Antonio, TX: Psychological Corporation

[c60] Wechsler D. (1998a). WAIS–IIIUK administration and scoring manual. London, UK: Psychological Corporation

[c61] Wechsler D. (1998b). WMS–IIIUK administration and scoring manual. London, UK: Psychological Corporation

[c62] Wechsler D. (2001). Wechsler Test of Adult Reading: WTAR. New York, NY: Psychological Corporation

[c63] Widaman K. F., Ferrer E., & Conger R. D. (2010). Factorial invariance within longitudinal structural equation models: Measuring the same construct across time. Child Development Perspectives, 4, 10–18. doi:10.1111/j.1750-8606.2009.00110.x 20369028 PMC2848495

[c64] Wilson R. S., Barnes L. L., Mendes de Leon C. F., & Evans D. A. (2009). Cognition and survival in a biracial urban population of old people. Intelligence, 37, 545–550. doi:10.1016/j.intell.2008.10.002

[c65] Wilson R. S., Beckett L. A., Barnes L. L., Schneider J. A., Bach J., Evans D. A., & Bennett D. A. (2002). Individual differences in rates of change in cognitive abilities of older persons. Psychology and Aging, 17, 179–193. doi:10.1037/0882-7974.17.2.179 12061405

[c67] World Health Organization (2011). Use of glycated haemoglobin (HbA1c) in the diagnosis of diabetes mellitus. Retrieved from http://www.who.int/diabetes/publications/report-hba1c_2011.pdf 26158184

[c68] Zahodne L. B., Glymour M. M., Sparks C., Bontempo D., Dixon R. A., MacDonald S. W., & Manly J. J. (2011). Education does not slow cognitive decline with aging: 12-year evidence from the Victoria Longitudinal Study. Journal of the International Neuropsychological Society, 17, 1039–1046. doi:10.1017/S1355617711001044 21923980 PMC3285821

[c69] Zimprich D., & Martin M. (2002). Can longitudinal changes in processing speed explain longitudinal changes in fluid intelligence. Psychology and Aging, 17, 690–695. doi:10.1037/0882-7974.17.4.690 12507364

